# Dynamic Touch as Common Ground for Enactivism and Ecological Psychology

**DOI:** 10.3389/fpsyg.2020.01257

**Published:** 2020-06-10

**Authors:** David Travieso, Lorena Lobo, Carlos de Paz, Thijme E. Langelaar, Jorge Ibáñez-Gijón, David M. Jacobs

**Affiliations:** ^1^Facultad de Psicología, Universidad Autónoma de Madrid, Madrid, Spain; ^2^Facultad de Ciencias de la Salud y de la Educación, Universidad a Distancia de Madrid, Villalba, Spain; ^3^Embodied Cognitive Science Unit, Okinawa Institute of Science and Technology, Okinawa, Japan; ^4^Faculty of Medical Sciences, University of Groningen, Groningen, Netherlands

**Keywords:** ecological psychology, dynamic touch, enactivism, intentionality, postcognitivism

## Abstract

The main purpose of this article is to show that enactivism and ecological psychology share more aspects than is often recognized. Rather than debating about differences, commonalities between the approaches are illustrated with the example of dynamic touch. Dynamic touch is a form of touch that implies muscles and tendons and that allows the perception of hand-held objects that are wielded but not seen. Given that perceivers perform the wielding movements with effort, dynamic touch necessarily implies active exploration. The strength of dynamic touch as an example lies in the fact that it has been formalized and analyzed in detail at the level of the laws that govern the organism-environment system. The example provides empirically supported instantiations of sensorimotor contingencies, in enactivist terms, and of intentional exploration and information detection, in ecological terms. Moreover, dynamic touch is a practical example of the enactivist concepts of bringing-forth the world and sense-making. As a second purpose, we use the example of dynamic touch to clarify key concepts of the ecological approach. Specifically, we analyze the concepts of invariance and affordance, indicating the crucial difference between perceiving and actualizing affordances, and highlighting the importance of these concepts for the dialogue between enactivism and ecological psychology.

## Enactivism and Ecological Psychology Should Bridge the Uncanny Valley

The enactive and ecological approaches are two disciplines concerned with understanding cognitive systems using a perspective that avoids scientific constructs such as internal representations or mental states, focusing instead on the interaction between the agent and its environment. Despite these strong commonalities, confrontation between enactive and ecological approaches has been the norm since the early days of enactivism. In the introduction of their book, *Sensorimotor Life*, Di Paolo et al. (2017) consider the relation between the enactive approach to cognition and the ecological approach to perception and action. In their words:

The relation between the schools of thought is one of strange familiarity, as if their respective practitioners were staring at each other across an uncanny valley. It is true that both approaches overlap in their rejection of representationalism, but this does not mean they are necessarily rejecting the same thing. As we will discuss further in this chapter and demonstrate in the following ones, the enactive perspective rejects a functionalist general approach to cognition, whereas ecological psychology rejects the assumption of the poverty of environmental information. These are not the same thing. For Gibsonians, perception is still about information pickup, but not for enactivists, who conceive of perception as an aspect of sense-making, a concept that is explicitly grounded in the notion of autonomous agency. (Footnote 3, p. 18)

In this article, we unfold the concepts that, according to Di Paolo et al. (2017), divide the traditions. We consider what information pick-up is for ecological psychology and whether it is compatible with the ideas of sense-making and autonomous agency from the enactivist approach.

As discussed in our previous work (Higueras-Herbada et al., 2019), the so-called 4E approaches (Gonzalez-Grandon and Froese, 2018; cf. Calvo and Gomila, 2008) – which include enactivism – only turn an eye to the much older ecological approach occasionally. As do the 4E approaches, the ecological approach has criticized the cognitivist approach from its very beginning (see Fultot et al., 2016, and the commentaries on that article; see also Lobo et al., 2018, for a historical overview of the ecological approach). The different origins and foci of enactivism and ecological psychology, however, have at times prevented that the commonalities between the approaches are appropriately reflected in the writings of scholars of the respective approaches (cf. Baggs, 2018). As Di Paolo et al. (2017) state:

This is one of the reasons for the admittedly rather quick dismissal of ecological psychology by Varela et al. (1991), who saw it only capable of providing a theory of cognition on the side of the environment. In later years, there have been many attempts at bringing the two traditions closer to each other. (Footnote 3, p. 18)

As indicated in the previous quote, recently there have been a number of attempts to relate enactivism and ecological psychology, giving room to at least three main positions (Higueras-Herbada et al., 2019). The first position considers the approaches irreconcilable (Cariani, 2016) or with substantial differences (Mossio and Taraborelli, 2008). The second position considers the approaches complementary, focusing on different levels of analysis. Thus, whereas the ecological theory of perception focuses on the ecological level of analysis, enactivism focuses on autonomous agency or on a subpersonal level of analysis (Heras-Escribano, 2016, 2019a,b; McGann, 2016). A third position, as formulated by Stapleton (2016) in her commentary on Fultot et al. (2016), holds that there are already examples that joint crucial concepts of the considered approaches:

It seems to me that much of the research developed in the ecological psychology approach, and the conceptual tools used, are valuably incorporated by enactivists to flesh out a full framework of life and mind. Likewise, ecological psychology can benefit from the depth of the enactivist enterprise. (p. 327)

We believe that theoretical papers concerning the enactive and ecological approaches should be understood as being engaged in dialogical discussions (Voloshinov and Bachtin, 1986). In such discussions, main concepts are treated as *dialogemes*, which is to say, as voices where the content of the terms has to be colonized by a tradition. In such a process, differences between approaches are often stressed, even subtle ones, concealing strong commonalities. In order to avoid such detrimental drift, the present article takes the opposite direction and aims to stress commonalities.

Just above the quote that opened this article, Di Paolo et al. (2017) state:

As proof of how close the approaches can be in concrete cases, we make use throughout this book of work originating in the Gibsonian tradition (Gibson, 1979/1986). This tradition usually supplies some of the clearest examples of how dynamical engagements and bodily synergies can be explanatorily powerful. (Footnote 3, p.18)

In this article, we focus on what we consider the best example to indicate points of coincidence of the approaches, and to describe classic ecological concepts such as affordance, invariant, and information pick-up through intentional (goal-driven) exploration. This example is the one of dynamic touch.

## What Is Dynamic Touch?

As far as we are aware, the study of dynamic touch can be traced back to Gibson’s article *Observations on Active Touch* (Gibson, 1962) and his seminal book *The Senses Considered as Perceptual Systems* (Gibson, 1966). In the article, Gibson questioned the everyday relevance of touch as conceived as a passive sensory channel. Instead, he highlighted the active nature of touch and the prominent role of exploration. He did so by comparing passive and active conditions of haptic perception in a series of experiments. In his words:

Active touch refers to what is ordinarily called *touching*. This ought to be distinguished from passive touch, or *being touched*. In one case the impression on the skin is brought about by the perceiver himself and in the other case by some outside agency …. Active touch is an exploratory rather than a merely receptive sense. When a person touches something with his fingers he produces the stimulation as it were. More exactly, variations in skin stimulation are caused by variation in his motor activity. … Such movements are not the ordinary kind usually thought of as responses. They do not modify the environment but only the stimuli coming from the environment. (Gibson, 1962, p. 477)

Gibson referred for the first time to dynamic touch (dynamic touching in the original) in the two chapters about haptics in his 1966 book (Gibson, 1966). In these chapters, Gibson recognized that part of the kinesthetic system allows a muscle-based perception of properties of external objects (Gibson, 1966, p.109). This is why dynamic touch, on occasions, is referred to as muscle-based perception.

In a practical sense, dynamic touch concerns the everyday haptic perception of natural and manufactured objects that we hold in our hands. Just to name a few examples, this includes the perception of how far we can throw objects through hefting (Bingham et al., 1989; Zhu and Bingham, 2008, 2010), if objects can be used for hammering (Wagman and Carello, 2001), the perception of shape (Burton et al., 1990), tool use (Michaels et al., 2007), the perception of heaviness (Shockley et al., 2001), and even an explanation of the size-weight illusion (Amazeen and Turvey, 1996).

Manipulating objects, such as a tennis racket, produces perceptions that cannot be explained appealing exclusively to the stimulation of the skin. In Gibson’s (1966) words:

The passive skin can be stimulated by an object resting on it, the amount of pressure (that is, skin deformation) being proportional to the weight of the object, but in this case discrimination is rather poor. It is much better when the object is lifted. (p. 127)

The different amounts of stretching and contracting of muscles, as well as the forces exerted by and on the muscles and on the tendons, change depending on the movements that are performed and on the form, size, and mass distribution of the object. Those variations form the main sensory basis of dynamic touch (Carello and Turvey, 2015, 2017).

The pivotal elements of the ecological conception of dynamic touch are the laws that connect motor components (forces exerted by muscles) and sensory components (in muscles and tendons). In this regard, the ecological portrayal of dynamic touch shows remarkable similarities with key concepts of the enactive tradition. Consider the example of the softness of a sponge that figures importantly in the writings of enactive scholars. According to O’Regan et al. (2005):

Having the sensation of softness consists in being aware that one can exercise certain practical skills with respect to the sponge: one can, for example, press it, and it will yield under the pressure. The experience of softness of the sponge is characterized by a variety of such possible patterns of interaction with the sponge, and the laws that describe these sensorimotor interactions we call, following MacKay (1962), laws of sensorimotor contingency (O’Regan and Noë, 2001). (p. 56)

In this regard it is curious to note that, when Gibson developed his intuitions about the functioning of dynamic touch, he cited several studies on the perception of softness, much in line with the writings of enactivist scholars. In Gibson’s (1966) words:

The firmness or softness of a material substance is a property of the substance that is registered when forces are exerted on it by the hand. Scott-Blair and Coppen (Harper, 1952) investigated the perception of the firmness-softness of industrial substances (rubber, bitumen) by having the observer squeeze a cylindrical sample of the material to be graded. The “feel” of the material was quite clear at the end of this dynamic action. They concluded that the perception “had the nature of a Gestalt,” but I would suggest instead that an invariant was isolated. (p. 128)

Gibson (1966) further suggested that the invariant involved in the perception of softness relates to the ratio of the force exerted and the amount of depression of the surface (cf. Harper and Stevens, 1964). In the case of dynamic touch, the relevant invariants relate forces to movements. Let us now introduce the physics of these invariants.

## The Laws That Govern the Organism-Environment Interaction in Dynamic Touch

Early research on dynamic touch analyzed the mechanical properties that govern the perception of the length of hand-held rods. In their pioneering study, Solomon and Turvey (1988) reported a linear relation between actual and perceived length and they argued that the observed pattern of results was consistent with the claim that the length judgments were based on the mechanical property that is referred to as *first principal moment of inertia* (cf. Kreifeldt and Chuang, 1979). Although this claim has been supported by later studies (e.g., Fitzpatrick et al., 1994), it has also been questioned (e.g., Kingma et al., 2004). Kingma et al. demonstrated the importance of a different mechanical property, referred to as *static moment* (cf. van de Langenberg et al., 2006). In the present section, we define such mechanical properties and provide intuitions about how these properties determine the relations between exerted forces and resulting movements.

Consider a rod that is loosely attached to a support at one of its ends so that the force of gravity orients the rod with its longitudinal axis toward the ground. Measuring the force exerted by the rod is one of the ways that allows the detection of the mechanical property *mass*. To facilitate the presentation of less well-known properties later in this section, it may be helpful to note that the mass (*M*) of an object can approximated by:

(1) $$M=\sum_{i=1}^{N}m_{i},$$

in which the *m_*i*_s* represent the point-masses of the object at *N* different points (integral-form versions of this equation and of Eqs 2 and 3 can be found in Jacobs et al., 2009). As captured by Newton’s second law of motion (*force = mass × acceleration*), mass can also be defined as the resistance of the object to linear accelerations. This means that moving a rod in a direction that is aligned with its longitudinal axis may reveal mass as the invariant that relates force and acceleration.

The equation that defines mass does not include a term that relates to the length of the object. This means that one cannot differentiate rods with equal mass and different lengths with linear movements along the longitudinal axis. Analogously, an object can be attached to ropes of different lengths and if one holds the rope vertically, one can detect mass, but not the length of the rope. If, however, all the rods that one encounters are of the same material and diameter (for instance in a laboratory situation), then length and mass co-vary perfectly. This means that, for such a particular set of rods, exploratory movements that allow the detection of mass also allow the detection of length.

Now consider a rod that is held by its end and maintained with its longitudinal axis horizontal to the ground. The rotational force that is exerted by the rod at the point of rotation corresponds to the mechanical property called static moment. Extending Equation 1, static moment (*SM*) can be defined as:

(2) $$S\,M=\sum_{i=1}^{N}m_{i}\,d_{i},$$

in which each *d*_*i*_ is the distance of the corresponding point-mass *m*_*i*_ to the axis of rotation. For rods with the same mass, homogeneously distributed over the length of the rod, the static moment is higher for longer rods. Therefore, under certain laboratory circumstances, statically maintaining a rod as described above may allow one to differentiate the lengths of objects with the same mass.

**FIGURE 1:**
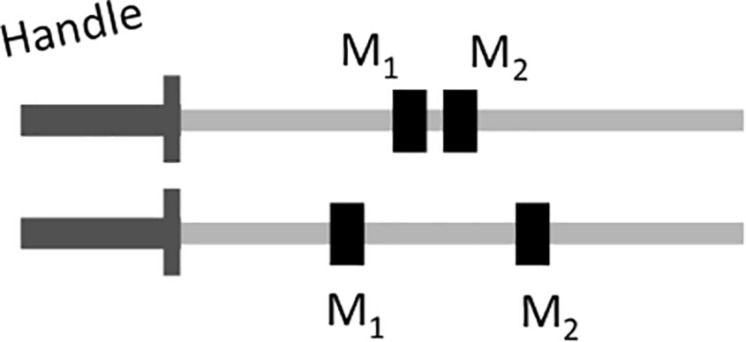
Two rods with equal mass and static moment but with different principal moments of inertia.

As a next step to introduce the mechanical properties and laws that are most relevant to dynamic touch, we illustrate how rods can be modified without changing their mass or static moment. Consider Figure 1. The rods have the same mass. They also have the same static moment, and thus exert the same rotational force when held still horizontally. However, if one actively rotates the rods, the lower one offers more resistance. This is so because the resistance to rotation offered by mass is a squared function of distance; hence, whereas moving *M*_2_ away from the axis of rotation is compensated by moving *M*_1_ nearer for static moment, this is not the case for the resistance against rotation. More precisely, for one-dimensional rotations, the moment of inertia (*I*) can be defined as:

(3) $$I=\sum_{i=1}^{N}m_{i}\,d_{i}^{2}.$$

In analogy to Newton’s second law for linear motion, the moment of inertia determines the acceleration that is obtained with a rotational force (i.e., *τ = I ×* α), meaning that the moment of inertia can be measured as the invariant relation between rotational acceleration, α, and force, *τ*.

As a final step in our introduction to the relevant mechanical properties and laws, one should note that, so far, we have only considered one-dimensional rotations. Even if one considers a single point of rotation, however, objects can be rotated around different axes, leading to different amounts of resistance. A full description of the 3-dimensional resistance to rotation that is offered by an object is provided by the inertia tensor, which is a symmetric 3 × 3 matrix with nine inertial components. The inertia tensor,***I***, relates three-dimensional forces to three-dimensional accelerations according to the formula: ***τ = I ×* α**, in which the use of boldface indicates the fact that the symbols stand for vector and matrix properties. If one diagonalizes the inertia tensor, the eigenvalues that one obtains are the mechanical properties referred to as the first, second, and third principal moments of inertia (*I*_1_ to *I*_3_). These moments can also be described as the resistance against rotation around three orthogonal axes that are ordered from the highest amount of resistance to rotation to the lowest amount of resistance. For a symmetric rod, *I*_1_ and *I*_2_ are identical and correspond to rotation around axes that are orthogonal to the longitudinal axis of the rod, and *I*_3_ corresponds to rotation around the longitudinal axis.

It is easy to intuitively experiment with some of these mechanical properties and laws. To do so, we ask you to hold a small rod between the thumb and index finger (for instance a wooden ruler or any other elongated object). The rod will move until aligned longitudinally with gravity. Move it up and down, so that you can feel its mass. But, what about its length? Then, keep it in a horizontal position. Does the effort change? What about mass and length perception? Finally, grip the object from the center and rotate it like a seesaw, and do the same while holding it by the end (rotations with low and high *I*_1_, respectively). This small experiment provides an intuitive demonstration that the relation between self-produced forces and resulting accelerations allows us to detect properties of wielded objects.

To summarize this section, during wielding, different types of forces stand in different invariant relations to the resulting accelerations. An enactivist may call these relations *sensorimotor laws*. Being sensitive to these relations, or sensorimotor laws, allows the perceiver to detect the mechanical properties that underlie, or determine, the invariant relations. The starting point of perceiving through dynamic touch, however, is not formed by the mechanical properties that underlie the agent-environment interactions. Instead, the starting point is formed by the exploratory movements exerted by an intentional agent. The next section, therefore, analyzes the exploratory movements and the role of intention as modifier of these movements.

## Intentional Exploration: Bringing-Forth the World Through Dynamic Touch

The concept of bringing-forth the world may be attributed to Varela et al. (1991), who write: “Cognition in its most encompassing sense consists in the enactment or *a bringing forth of a world* [emphasis added] by a viable history of structural coupling.” (p. 205). Elaborating on the concept, Proulx (2008) states:

It is my structure that allows me to “see” or perceive things in the physical world, and so my structure allows me to give meaning to the attributes of the physical world. I – my structure – allow the physical world to be brought forth. If these attributes of the physical world are outside of my structure, outside of my capacity to make sense of them, I cannot distinguish them and cannot perceive them. In other words, they cannot “trigger” anything in me. Hence, I bring forth the physical world’s attributes when I give/create meaning to it – I acknowledge their physical “presence” by bringing them forth. If I do not bring them forth, the physical world’s attributes will still be “there,” but they will remain unnoticed, not made sense of and kept “in the dark.” It is in this sense that the physical attributes themselves are brought forth by my interaction with them (if I perceive them). In some sense, I make the physical world emerge. (p. 21–22)

In our effort to stress commonalities between the enactivist and ecological approaches, we should note the similarities between the intentional exploration in dynamic touch and the enactivist concept of bringing-forth the world. The mechanical properties defined in the previous section (mass, static moment, inertia tensor) are intrinsic properties that can be detected only by acting upon them. Although the inertia tensor of an object can physically be described independently of a perceiver, in order to detect or measure the property, perceivers apply forces on the object and identify relational aspects of the emerging interaction.

The fact that, in dynamic touch, the perceiver chooses which mechanical properties to act upon, or bring forth, brings us to the concept of intentionality. A common portrayal of the ecological approach from enactive scholars considers that action is reduced to mere movement, thus leaving intentionality out of the explanations. For example, Di Paolo (2016) states:

In addition to a deep understanding of the environment, we need a theory that pays attention to the perceiver as an active agent and her capacity to engage her world meaningfully. “Active” here does not mean simply “moving” (this is well-covered in ecological psychology). It means engaged in a regulated coupling with the environment, generating goals and pursuing them, moving in ways that alter the constraints that link the agent and the environment as coupled systems. (p. 327)

The example of dynamic touch, however, shows the central role of actions and intentions in the ecological approach to perception. In the ecological portrayal of dynamic touch, it is particularly clear that exploratory movements are performed *because* the perceiver intentionally, or purposefully, aims to reveal certain mechanical properties of the object while ignoring others. This notion of the role of action and intention in perception is shared with enactivists researchers. A prominent example can be found in the book *Action in Perception* (Noë, 2004):

Think of a blind person tip-tapping his or her way around a cluttered space, perceiving that space by touch, not all at once, but through time, by skillful probing and movement. This is, or at least ought to be, our paradigm of what perceiving is. The world makes itself available to the perceiver through physical movement and interaction. (p. 1)

The concept of intentionality was, in fact, one of the key concepts in the pioneering research on dynamic touch by Solomon and Turvey (1988), and thereby one of the foundational concepts for the body of research on dynamic touch. In their words:

With a haptic subsystem, intentionality identifies the goal to be attained. On the basis of the intention of the subject/performer, the parts of the haptic perceptual system are assembled into a special-purpose machine capable of attaining the desired goal …. when the muscles and other tissues are assembled into a subsystem, a functional unit, the behavior of the subsystem is conjugate to the properties of the object under exploration. (Solomon and Turvey, 1988, p. 405)

Later in this section we describe empirical research that demonstrates how different intentions lead to different exploratory movements. First, however, we describe empirical research that indicates how different exploratory movements determine the mechanical properties that are detected.

Burton and Turvey (1990) performed experiments on the perception of length through dynamic touch with unrestricted movements as well as with the instruction not to move the rod. With instructional restrictions on the movements, static moment predicted the length judgments better than the first principal moment of inertia, whereas this was the other way around for unrestricted movements. Relatedly, in their study on length perception through dynamic touch, Lobo and Travieso (2012) systematically restricted the exploratory movements in six movement conditions with different amplitudes and frequencies. With faster movements, more accurate judgments were obtained, supporting the idea that, in unrestricted conditions, length perception relies on the first principal moment of inertia.

**FIGURE 2:**
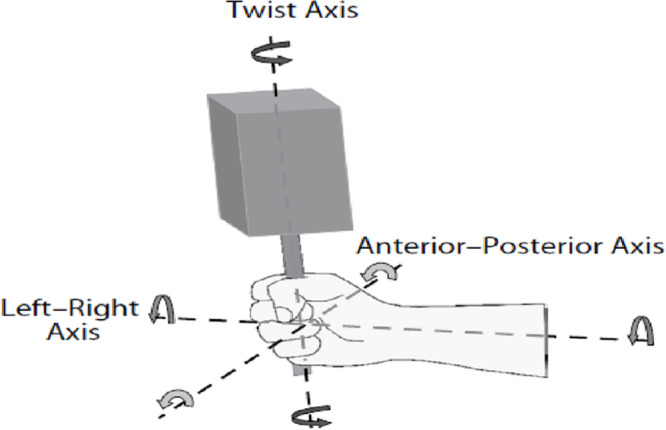
Type of objects used in the experiments by Arzamarski et al. (2010) with the relevant axes of rotation. Figure 8 from Arzamarski et al. (2010).

Similar conclusions concerning the importance of exploratory movements were obtained by Harrison et al. (2011). These authors also showed that the intention to detect different affordances (e.g., holdability, rotatability) affected length perception. Their results “revealed perception to be constrained by (a) the moments of mass distribution of the hand-tool system, (b) the qualities of exploratory wielding movements, and (c) the intention to perceive each specific property” (Harrison et al., 2011, p. 193). A further example of how intentions affect the detected mechanical properties as well as the exploratory movements can be found in Arzamarski et al. (2010; cf. Riley et al., 2002). These authors manipulated the intention of participants by asking them to report either the length or the width of hand-held objects. When participants intended to perceive length, the exploratory movements showed relatively more rotation around the anterior-posterior axis (as defined in Figure 2), and the judgments were more closely related to *I*_1_ than to *I*_3_. On the contrary, when the intention was to perceive width, participants performed relatively more rotations around the twist axis and estimations were more closely related to *I*_3_ than to *I*_1_.

Rather than thinking about relatively simple exploratory movements such as pure rotations or twists, one may prefer to think about more complex ones. It is interesting to indicate in this regard that Stephen et al. (2010) argue that fractal fluctuations in exploration reveal the detection of information at many time-scales:

Indeed, even the miniscule musculoskeletal fluctuations incident to static holding have been claimed to inform haptic judgments (Burton and Turvey, 1990). Whereas static holding is simply the maintenance of balance among muscular and gravitational forces, wielding signifies a strongly intentional excursion from balance that is meant to produce new or richer information about the inertial properties of the object. Of course, active forces in wielding movements will still interact with reactive forces: The inertial properties of the object still exert effects on the trajectory of the limb that are not explicitly prescribed by the muscle forces exerted during wielding. (p. 2)

As an aside, the idea of multi-scale behavior is reminiscent to an ecological theory of learning, referred to as *direct learning* (Jacobs and Michaels, 2007), which holds that phenomena at the time-scale of learning can be explained with the ecological principles developed for the time-scale of perceiving and acting. The above-mentioned study of Arzamarski et al. (2010) is related to this theory, as well as other learning studies in the field of dynamic touch (Michaels et al., 2008; Jacobs et al., 2009; Withagen and van Wermeskerken, 2009). We refer the reader to Higueras-Herbada et al. (2019) for a description of that theory and its relevance to the dialogue between the enactive and ecological approaches.

To summarize the gist of this section, the type of exploratory movements that are performed determine which mechanical properties become determinants of the agent-environment interaction and which mechanical properties are irrelevant for that interaction (e.g., twisting movements are affected by *I*_3_ but not by *I*_1_). The ecological portrayal of dynamic touch, therefore, starts with the intention to perceive some property. This intention leads to exploratory behavior that is determined by, and hence reveals in the interaction, those mechanical properties that relate to the property that the agent intends to perceive. This portrayal is aligned with the writing of many enactivists, who may say that the purposeful, autonomous agent *brings forth* those mechanical properties that are relevant to her goals, leaving in the dark other properties.

## Affordances and Invariants in Dynamic Touch

Our next goal is to illustrate and hopefully clarify two key concepts from the ecological approach: affordances and invariants. Instead of providing canonical definitions of these terms, we consider them using the example of dynamic touch. With respect to affordances, we focus on the distinction between perceiving and actualizing, which, we believe, is often under-emphasized or misunderstood in the writings of scholars that compare enactivism and ecological psychology.

When Gibson (1966) introduced the concept of invariant in dynamic touch, he stated the following:

The mass of an object can be judged, in fact, by wielding it in any of a variety of ways, such as tossing and catching, or shaking it from side to side. One can only conclude that the judgment is based on information, not on the sensations. The stimulus information from wielding can only be an invariant of the changing flux of stimulation in the muscles and tendons, an exterospecific invariant in this play of forces. Whatever specifies the mass of the object presumably can be isolated from the change, and the wielding of the object has the function of separating off the permanent component from the changes. The merely propriospecific information can thus be filtered out, as it were, leaving pure information about the object. This process takes time, for an invariant can only emerge from a series of transformations over time. (p. 127)

When we wield a tennis racket, for example, rotational hand and arm forces lead to different movements. Those movements depend on the original position of the hand-arm system and the racket, and on the dynamics of the whole system in inertial terms. The invariants that reside in the flux of change of forces and movements are specific to intrinsic properties of the wielded objects. However, in contrast to the interpretation of ecological psychology as a physicalist approach, the information that is used for perception through dynamic touch is not the specification of such mechanical properties in discrete stimuli that are internalized, or values that perceivers infer from stimulation that is imposed on them. Quite the opposite, the invariants exist because of the laws that govern the organism-environment system only when these laws are combined with the intentional exploration that creates the necessary change for the invariants to come into existence. Perceiving, then, is the detection of the invariants in the organism-environment interaction that specify possibilities of action. As ecological psychologists would say: we detect invariants and we perceive affordances.

As Carello and Turvey (2017) remind us, Gibson (1966) first introduced the ideas behind the concept of affordance in his reflections on dynamic touch:

Although he did not develop his notion of affordance until his 1979/1986 book it is, in fact, mentioned in Gibson (1966) in one of the chapters on the haptic system. In arguing against the tradition of imposing discrete stimuli on passive participants who were to report “awareness of the impression, not the object making it” (p. 98), he recommended that an active observer obtaining patterns of stimulation should be “allowed to report what he perceives, including what it affords or might be used for” (p. 99). (Carello and Turvey, 2017, p. 97)

For rods, examples of affordances may be throwability, holdability, rotatability, whether other objects can be reached with the rods, or whether the rods are suitable for certain hitting actions. In contrast to other properties (e.g., the length or shape of rods), affordances are defined with respect to the organism’s purposeful actions. This means taking the organism-environment system as the unit of explanation. Thus, when wielding an object and producing rotations, perceivers detect the relation between exerted forces and resulting movements, perceiving affordances that allow them to prospectively control further movements (e.g., Bongers et al., 2004).

We believe that it is crucial for the dialogue between enactivism and ecological psychology to clarify that perceiving an affordance is not the same as actualizing an affordance (i.e., performing the action that is afforded; Michaels et al., 2001). For instance, perceiving that a stone affords throwing (Bingham et al., 1989) is not the same as throwing the stone, or perceiving that a ball can be caught is not the same as actually catching the ball (Jacobs and Michaels, 2006). Consider a further example. During the night, a person wakes up and wants to go to the bathroom without turning the lights on. Instead, she uses the light of her phone to illuminate her peripersonal space (the space immediately close to her). With this light, she sees one slipper under the bed, but, unfortunately, a bit far, so she cannot reach it with her arm. She stares at the slipper as she taps nearby objects in an attempt to find something that allows her to reach the slipper. She grabs an object and wields it, but rapidly discards it, as it is too short. Then she grabs another object, does the same quick wielding, and finally uses it, knowing that it will be long enough to reach the slipper. Using the ecological vocabulary, one may say that the sequence of actions started with the intention to get the missing slipper. Using dynamic touch, in the first try with an object she did not perceive the affordance of reaching the slipper. In the second try, she did perceive the affordance. After perceiving the affordance, she actualized the affordance by actually reaching toward the slipper.

When actualizing an affordance, then, such as throwing a stone, catching a ball, or reaching for a slipper, information is used that allows the actor to control the relevant forces. The action itself emerges from the information-action couplings and the dynamics of the agent-environment system. This means that, for the actualization of affordances, one does not need environmental information that specifies the affordance. Hence, in line with what is often claimed by enactivists (e.g., Varela et al., 1991), for a theory of perceptually-guided action (i.e., a theory about actualizing affordances), one does not need environmental information that specifies affordances.

In order to veridically perceive an affordance, however, often done before actualizing the affordance, a relation of specificity must be maintained between the agent and the environment (Jacobs and Michaels, 2002). In the case of dynamic touch, this implies a lawful chain of specificity that goes from the intention to perceive the affordance, to invariants in the flux of forces and movements that is created during the exploration, via the mechanical properties of the wielded object, to the actual affordance, as well as vice versa. If an affordance is not specified in detectable information, it cannot be perceived. Ecological psychologists are interested in the perception of affordances, for which the specification of affordances is indispensable, as well as in the actualization of affordances, which may be analyzed with less concern about what is specified by the information that guides the movement (cf. Michaels et al., 2001).

Varela et al. (1991), in their treatment of the ecological approach, had in mind the actualization of affordances when they describe their own position. This is clear, for example, when they write: “The overall concern of an enactive approach to perception is … to determine the common principles or lawful linkages between sensory and motor systems that explain how action can be perceptually guided” (p. 173). In this regard, the enactive approach is more closely related to the part of the ecological approach about the actualization of affordances than to the part about the perception of affordances. Rather than noting existing commonalities with ecological research about the actualization of affordances, however, Varela et al. (1991) distanciate themselves from the ecological approach by referring to ecological ideas concerning the perception of affordances: “In Gibson’s view … perceptually guided action consists in ‘picking up’ or ‘attending to’ invariances in the ambient light that directly specify their environmental source” (p. 203). This quote seems to concern the actualization of affordances (“perceptually guided action”) in its first part but changes its emphasis toward the perception of affordances (“invariants that specify their source”) in the latter part. To summarize, rather than searching for commonalities with theoretical and empirical research from the ecological tradition about the actualization of affordances, enactivist scholars often choose to stress differences by focusing on parts of the ecological approach that concern the perception of affordances – an issue that, one way or another, any approach has to address.

Apart from the confusion concerning perception and actualization, the concept of affordance is often claimed to be well suited to the enactivist approach (Varela et al., 1991) and is mentioned ubiquitously by enactivists (e.g., Noë, 2004; Di Paolo et al., 2017; Gallagher, 2017). This is so because affordances are related to the bringing-forth the world concept of enactivism and to sense-making. We briefly address the concept of sense-making before our concluding section.

## Sense-Making Agency in Dynamic Touch

Sense-making agency refers to a conception of agency by which the organism builds up its agency through meaningful interactions with the environment. In this context, perception is not a blind contact with the environment that imprints its effect on the sensory surfaces, which is afterward processed searching for regularities. Using the words of Thompson and Stapleton (2009):

Even the simplest organisms regulate their interactions with the world in such a way that they transform the world into a place of salience, meaning, and value – into an environment (Umwelt) in the proper biological sense of the term. This transformation of the world into an environment happens through the organism’s sense-making activity. (p. 25)

The concept of sense-making refers to the particular behavior of the autonomous organism that is determined by its structure (for example, only animals with eyes can see), which makes available certain properties of the physical world that constitute the environment for her. Moreover, enactivists remain skeptical of those animal-environment relations in which specification does not imply an intentional action on the part of the organism. A clear example is the detection of time-to-contact (Lee, 1976). Despite the fact that the original analysis concerned the active control of movement by the perceiver (i.e., the control of braking by a driver), the optical specification of time-to-contact can be demonstrated for the case where the perceiver actively approaches a static object as well as for the case where an object approaches a static perceiver. Thus, Di Paolo (2016) states that for enactivists:

Meaning is not just something that pops up in the relation between organism and environment. It necessitates a particular kind of activity on the part of the agent to emerge, i.e., sense-making. (p. 328)

In the paradigmatic case of dynamic touch, the history of interaction with the object consists of the exploratory wielding, where forces exerted by the agent produce changes in the position of the objects. The objects that are wielded during dynamic touch, and the mechanical properties that we described earlier in this article, therefore form part of the meaningful environment of the perceiving agent. An enactivist may claim that dynamic touch is the sense-making activity that makes that the rods and the relevant mechanical properties form part of the meaningful environment of the agent. The ecological example of dynamic touch, therefore, as well as the concept of affordance, can easily be related to enactivist concerns about sense-making. For further debate about the notion of sense-making we refer the reader to Fultot et al. (2016).

From our perspective, at the same time that the sense-making activity determines the ecological niche for the organism – only animals with eyes can see – the environment exerts selective pressure on the organisms, establishing constraints on the structure of the organism – eyes are adaptive only in an environment with light that obeys the laws of optics. Therefore, we can consider that the sense-making activity is determined by the structure of the organism, and the laws governing the sensorimotor activity are those describing the physical world in which they are embedded. The strength of the dynamic touch example is that, together with the sense-making activity, we have a detailed description of the laws governing the physical interaction. Borrowing, again, the words of Di Paolo (2016):

Despite the accent on agency as always situated in an environment, it is correct that there is a dearth of enactive theorizing about the environment, as noted by McGann (2014). Does this mean that such theorizing is unwanted or that it could not fit well with other enactive ideas? I do not think so. It is more a case that it has not been done yet, and what better encouragement to do this than to engage in ongoing dialogues with ecological psychologists. (p. 329)

## Conclusion

In this article we have aimed to reveal commonalities rather than emphasizing differences between enactivism and ecological psychology, using dynamic touch as example. We have argued that more similarities can be identified than is often indicated in the literature. In this sense, we have shown how dynamic touch can provide an excellent test bed for enactivist ideas concerning sensorimotor laws, bringing forth the world, goal-directed agency, and sense-making activity. In particular, the active and intentional information detection put forward by ecological psychologists implies that ecological information is not instructive, meaning that the information does not determine what is perceived or acted upon.

Given that not all perceptual tasks reveal such an obvious intentional exploration as dynamic touch, however, one may wonder whether dynamic touch can be considered a generalizable example. That is, would the enactivist and ecological approaches show as much similarity if analyzed with another example? In this regard, it is interesting to note that both enactivists and ecological psychologists tend to indicate similarities between vision and touch. To illustrate this for ecological psychology, consider two quotes from Gibson’s (1962) seminal article on active touch. Concerning passive touch and vision, he states:

In passive touch the individual makes no voluntary movements. Similarly, in passive vision he makes no eye movements, which means that he must voluntarily fixate his eyes on a point specified by the experimenter. Neither state is natural to an individual. (Gibson, 1962, p. 489)

Concerning active touch and vision, in contrast, he states:

The foregoing survey suggests that vision and touch have nothing in common only when they are conceived as channels for pure and meaningless sensory data. When they are conceived instead as channels for information-pickup, having active and exploratory sense organs, they have much in common. (Gibson, 1962, p. 490)

To illustrate that similarities between vision and touch are also indicated in the work of enactive scholars, reflecting a notion of perception in which the enactive and ecological approaches can meet, we conclude with a quote from a prominent predecessor of the enactive approach, Maurice Merleau-Ponty: “Vision is a palpation with the look” (cited in Noë, 2004, p. 35).

## Author Contributions

DT wrote the initial draft of the manuscript. All authors contributed to the planification, writing, and correction of the manuscript.

## Conflict of Interest

The authors declare that the research was conducted in the absence of any commercial or financial relationships that could be construed as a potential conflict of interest.

## References

[B1] Amazeen E. L. Turvey M. T. (1996). Weight perception and the haptic size–weight illusion are functions of the inertia tensor. *J. Exp. Psychol. Human* 22 213–232. 10.1037/0096-1523.22.1.213 8742263

[B2] Arzamarski R. Isenhower R. W. Kay B. A. Turvey M. T. Michaels C. F. (2010). Effects of intention and learning on attention to information in dynamic touch. *Atten. Percept. Psycho.* 72 721–735. 10.3758/app.72.3.721 20348578

[B3] Baggs E. (2018). A psychology of the in between? *Constr. Found.* 133 395–397.

[B4] Bingham G. P. Schmidt R. C. Rosenblum L. D. (1989). Hefting for a maximum distance throw: a smart perceptual mechanism. *J. Exp. Psychol. Human* 15 507–528. 10.1037/0096-1523.15.3.507 2527959

[B5] Bongers R. M. Michaels C. F. Smitsman A. W. (2004). Variations of tool and task characteristics reveal that tool-use postures are anticipated. *J. Motor Behav.* 36 305–315. 10.3200/jmbr.36.3.305-315 15262626

[B6] Burton G. Turvey M. T. (1990). Perceiving the lengths of rods that are held but not wielded. *Ecol. Psychol.* 2 295–324. 10.1207/s15326969eco0204_1

[B7] Burton G. Turvey M. T. Solomon H. Y. (1990). Can shape be perceived by dynamic touch? *Percept. Psychophys.* 48 477–487. 10.3758/bf03211592 2247331

[B8] Calvo P. Gomila T. (2008). *Handbook of Cognitive Science: An Embodied Approach.* London: Elsevier.

[B9] Carello C. Turvey M. T. (2015). “Dynamic (effortful) touch,” in *Scholarpedia of Touch*, Vol. 10 eds Prescott T. J. Ahissar E. (Paris: Atlantis Press), 8242 10.4249/scholarpedia.8242

[B10] Carello C. Turvey M. T. (2017). Useful dimensions of haptic perception: 50 years after the senses considered as perceptual systems. *Ecol. Psychol.* 29 95–121. 10.1080/10407413.2017.1297188

[B11] Cariani P. A. (2016). Learning of new percept-action mappings is a constructive process of goal-directed self-modification. *Constr. Found.* 112 322–324.

[B12] Di Paolo E. (2016). Across the uncanny valley: the ecological, the enactive, and the strangely familiar. *Constr. Found.* 11 327–329.

[B13] Di Paolo E. Buhrmann T. Barandiaran X. (2017). *Sensorimotor Life: An Enactive Proposal.* New York, NY: Oxford University Press.

[B14] Fitzpatrick P. Carello C. Turvey M. T. (1994). Eigenvalues of the inertia tensor and exteroception by the “muscular sense”. *Neuroscience* 60 551–568. 10.1016/0306-4522(94)90264-x 8072695

[B15] Fultot M. Nie L. Carello C. (2016). Perception-action mutuality obviates mental construction. *Constr. Found.* 11 298–307.

[B16] Gallagher S. (2017). *Enactivist Interventions: Rethinking the Mind.* Oxford: Oxford University Press.

[B17] Gibson J. J. (1962). Observations on active touch. *Psychol. Rev.* 69 477–491. 10.1037/h0046962 13947730

[B18] Gibson J. J. (1966). *The Senses Considered as Perceptual Systems.* Hillsdale, NJ: Lawrence Erlbaum Associates.

[B19] Gibson J. J. (1979/1986). *The Ecological Approach to Visual Perception.* London: Routledge.

[B20] Gonzalez-Grandon X. Froese T. (2018). Grounding 4E cognition in Mexico: introduction to special issue on spotlight on 4E cognition research in Mexico. *Adapt. Behav.* 26 189–198. 10.1177/1059712318791633

[B21] Harper R. (1952). Physiological and psychological aspects of studies of craftsmanship in dairying. *Brit. J. Psychol. Monogr.* 28, 1–63.

[B22] Harper R. Stevens S. S. (1964). Subjective hardness of compliant materials. *Q. J. Exp. Psychol.* 16 204–215. 10.1080/17470216408416370

[B23] Harrison S. J. Hajnal A. Lopresti-Goodman S. Isenhower R. W. Kinsella-Shaw J. M. (2011). Perceiving action-relevant properties of tools through dynamic touch: effects of mass distribution, exploration style, and intention. *J. Exp. Psychol. Human* 37 193–206. 10.1037/a0020407 21077721

[B24] Heras-Escribano M. (2016). Embracing the environment: ecological answers for enactive problems. *Constr. Found.* 11 309–312.

[B25] Heras-Escribano M. (2019b). *The Philosophy of Affordances.* Cham: Palgrave Macmillan.

[B26] Heras-Escribano M. (2019a). Pragmatism, enactivism, and ecological psychology: towards a unified approach to post-cognitivism. *Synthese* 1–27. 10.1007/s11229-019-02111-1

[B27] Higueras-Herbada A. de Paz C. Jacobs D. M. Travieso D. Ibáñez-Gijón J. (2019). The direct learning theory: a naturalistic approach to learning for the post-cognitivist era. *Adapt. Behav.* 27 389–403. 10.1177/1059712319847136

[B28] Jacobs D. M. Michaels C. F. (2002). On the apparent paradox of learning and realism. *Ecol. Psychol.* 14 127–139. 10.1207/s15326969eco1403_2

[B29] Jacobs D. M. Michaels C. F. (2006). Lateral interception I: operative optical variables, attunement, and calibration. *J. Exp. Psychol. Human* 32 443–458. 10.1037/0096-1523.32.2.443 16634681

[B30] Jacobs D. M. Michaels C. F. (2007). Direct learning. *Ecol. Psychol.* 19 321–349. 10.1080/10407410701432337

[B31] Jacobs D. M. Silva P. L. Calvo J. (2009). An empirical illustration and formalization of the theory of direct learning: the muscle-based perception of kinetic properties. *Ecol. Psychol.* 21 245–289. 10.1080/10407410903058302

[B32] Kingma I. van de Langenberg R. Beek P. J. (2004). Which mechanical invariants are associated with the perception of length and heaviness of a nonvisible handheld rod? Testing the inertia tensor hypothesis. *J. Exp. Psychol. Human* 30 346–354. 10.1037/0096-1523.30.2.346 15053693

[B33] Kreifeldt J. G. Chuang M.-C. (1979). Moment of inertia: psychophysical study of an overlooked sensation. *Science* 206 588–590. 493965 10.1126/science.493965

[B34] Lee D. N. (1976). A theory of visual control of braking based on information about time-to-collision. *Perception* 5 437–459. 10.1068/p050437 1005020

[B35] Lobo L. Heras-Escribano M. Travieso D. (2018). The history and philosophy of ecological psychology. *Front. Psychol.* 9:2228. 10.3389/fpsyg.2018.02228 30555368 PMC6280920

[B36] Lobo L. Travieso D. (2012). El patrón de exploración modula la percepción de longitudes a través del tacto dinámico. *Psicothema* 24 55–61. 22269364

[B37] MacKay D. (1962). “Theoretical models of space perception,” in *Aspects of the Theory of Artificial Intelligence*, ed. Muses C. A. (New York, NY: Plenum Press), 10.1007/978-1-4899-6584-4_5

[B38] McGann M. (2014). Enacting a social ecology: radically embodied intersubjectivity. *Front. Psychol.* 5:1321. 10.3389/fpsyg.2014.01321 25477844 PMC4235264

[B39] McGann M. (2016). Enactivism and ecological psychology: divided by common ground. *Constr. Found.* 11 312–315.

[B40] Michaels C. F. Arzamarski R. Isenhower R. W. Jacobs D. M. (2008). Direct learning in dynamic touch. *J. Exp. Psychol. Human* 34:944. 10.1037/0096-1523.34.4.944 18665737

[B41] Michaels C. F. Weier Z. Harrison S. J. (2007). Using vision and dynamic touch to perceive the affordances of tools. *Perception* 36 750–772. 10.1068/p5593 17624120

[B42] Michaels C. F. Withagen R. Jacobs D. M. Zaal F. T. J. M. Bongers R. M. (2001). Information, perception, and action: a reply to commentators. *Ecol. Psychol.* 13 227–244. 10.1207/s15326969eco1303_3

[B43] Mossio M. Taraborelli D. (2008). Action-dependent perceptual invariants: from ecological to sensorimotor approaches. *Conscious. Cogn.* 17 1324–1340. 10.1016/j.concog.2007.12.003 18226924

[B44] Noë A. (2004). *Action in Perception.* Cambridge, MA: The MIT Press.

[B45] O’Regan J. K. Myin E. Noë A. (2005). Skill, corporality and alerting capacity in an account of sensory consciousness. *Prog. Brain Res.* 150 55–68. 10.1016/s0079-6123(05)50005-0 16186015

[B46] O’Regan J. K. Noë A. (2001). A sensorimotor account of vision and visual consciousness. *Behav. Brain Sci.* 24 939–973. 10.1017/s0140525x01000115 12239892

[B47] Proulx J. (2008). Some differences between Maturana and Varela’s theory of cognition and constructivism. *Complicity* 5 11–26. 10.29173/cmplct8778

[B48] Riley M. A. Wagman J. B. Santana M. V. Carello C. Turvey M. T. (2002). Perceptual behavior: recurrence analysis of a haptic exploratory procedure. *Perception* 31 481–510. 10.1068/p3176 12018792

[B49] Shockley K. Grocki M. Carello C. Turvey M. T. (2001). Somatosensory attunement to the rigid body laws. *Exp. Brain Res.* 136 133–137. 10.1007/s002210000589 11204408

[B50] Solomon H. Y. Turvey M. T. (1988). Haptically perceiving the distances reachable with hand-held objects. *J. Exp. Psychol. Human* 14 404–427. 10.1037/0096-1523.14.3.404 2971770

[B51] Stapleton M. (2016). Enactivism embraces ecological psychology. *Constr. Found.* 11 325–327.

[B52] Stephen D. G. Arzamarski R. Michaels C. F. (2010). The role of fractality in perceptual learning: exploration in dynamic touch. *J. Exp. Psychol. Human* 36 1161–1174. 10.1037/a0019219 20718566

[B53] Thompson E. Stapleton M. (2009). Making sense of sense-making: reflections on enactive and extended mind theories. *Topoi* 28 23–30. 10.31231/osf.io/3np9g

[B54] van de Langenberg R. Kingma I. Beek P. J. (2006). Mechanical invariants are implicated in dynamic touch as a function of their salience in the stimulus flow. *J. Exp. Psychol. Human* 32 1093–1106. 10.1037/0096-1523.32.5.1093 17002524

[B55] Varela F. J. Thompson E. Rosch E. (1991). *Embodied Mind: Cognitive Science and Human Experience.* Cambridge, MA: MIT Press.

[B56] Voloshinov V. N. Bachtin M. M. (1986). *Marxism and the Philosophy of Language.* Cambridge, MA: Harvard University Press.

[B57] Wagman J. B. Carello C. (2001). Affordances and inertial constraints on tool use. *Ecol. Psychol.* 13 173–195. 10.1207/s15326969eco1303_1

[B58] Withagen R. van Wermeskerken M. (2009). Individual differences in learning to perceive length by dynamic touch: evidence for variation in perceptual learning capacities. *Percept. Psychophys.* 71 64–75. 10.3758/app.71.1.64 19304597

[B59] Zhu Q. Bingham G. P. (2008). Is hefting to perceive the affordance for throwing a smart perceptual mechanism? *J. Exp. Psychol. Human* 34 929–943. 10.1037/0096-1523.34.4.929 18665736

[B60] Zhu Q. Bingham G. P. (2010). Learning to perceive the affordance for long-distance throwing: smart mechanism or function learning? *J. Exp. Psychol. Human* 36 862–875. 10.1037/a0018738 20695705

